# Supplementary material for: Evaluation and cost estimation of laboratory test overuse in 43 commonly ordered parameters through a Computerized Clinical Decision Support System (CCDSS) in a large university hospital
Source: PLoS One. 2020 Aug 6;15(8):e0237159. doi: 10.1371/journal.pone.0237159 (PMC7410244; doi:10.1371/journal.pone.0237159)
Supplement: Attachment — Submitted filename: Response to Reviewers.docx [file pone.0237159.s002.docx]

Dear editor,

I am thankful for your precious suggestions made to improve our work. They were carefully analysed and, as you can see from the attached reviewed document, taken into consideration and completely embraced.

On the basis of your suggestion, we reconsidered the whole paper and the meaning of “inappropriateness” and limited it only to the topic of “test overuse”. In “Discussion” section only comparisons with over-utilization rates of other authors have been included. Even the title has been changed in this light.

Although it is not feasible to apply appropriateness criteria and/or overutilization rules on all possible laboratory tests (over 1000), according to the other authors mentioned, we considered the most frequently performed tests and representative rules (applicable by automatic systems, considering a CCDSS use). We proceeded to specify this aspect in the “Setting” section (row 88).

Despite these limits (better explained in the “Strengths and limitations” section – row 194), the study collected data on a significant number of laboratory tests performed over a long period of time.

The common tests you missed probably fall into the “Complete Blood Count” panel (RBC, Hb, Ht, MCV, MCH, MCHC, RDW, PLTS). However, as you mentioned, we considered it appropriate to better specify the composition of the panel by adding a footnote to the tables and a sentence in the “Materials and methods” section (row 113).

Finally, we kindly ask you to include in the authors list Professor Maurizio Sanguinetti (President elect and Secretary General of ESCMID, Head of Department of Laboratory Sciences and Infectious Diseases of IRCCS Gemelli Hospital), for his fundamental contribution, as supervisor, in the final stages of this work.

Many thanks for your consideration.

Sincerely,

Dr Andrea Tamburrano

*Section of Hygiene - Institute of Public Health*

*Università Cattolica del Sacro Cuore di Roma*

Dear reviewer #1,

I am thankful for your precious revision made to improve our work. Your suggestions were carefully analysed and, as you can see from the attached reviewed document, taken into consideration and completely embraced.

We report point-by-point answers to your questions:

1) As you suggested, we proceeded to explain the context of the Italian National Health System by specifying in the “Analysis” section that all the treatments were carried out free of charge for the hospitalized patients; hospitalization costs (including laboratory tests) were reimbursed by the Regional Health System according to Medicare Diagnosis Related Groups (MS-DRGs) (row 132).

2) The origin of the requests and the samples is described in the “Setting” section (row 89): only requests from hospital internal departments were monitored.

3) As explained at Point 1, Italian NHS fully covers hospitalization costs according to MS-DRG. This is a flat cost and it’s linked to the main diagnosis and procedures performed during hospitalization. A reduction in inappropriate laboratory tests does not affect the reimbursement costs and generates net savings for the hospital.

4) Only medical staff can make exam requests in Italian Health System. We proceeded to better specify this information (row 91).

5) No temporary physicians were included in the analysis. Only internal staff could make exam requests through the CPOE login interface.

6) Although your suggestion is acceptable and very interesting, unfortunately, the Local Ethical Committee did not allow us to track information related to physician demography, it’s specific role and the department of origin.

7) As described in “Strengths and limitations” section (row 199) physicians were not alerted and the CCDSS worked silently, without blocking or generating pop-ups. In this way we were able to assess the rate of inappropriateness in laboratory tests overuse without the confounding factor of physician’s awareness of a control system on laboratory requests.

8 and 9) All the examined tests have been ordered and counted as single tests, except for “Complete blood count” in which a panel of tests was ordered and counted in bulk (all together). As you suggested, we better explained this aspect in “Computerized Clinical Decision Support System (CCDSS)” section (row 113). Also, a footnote to the two tables was added.

Following your suggestion, we have reduced the “Conclusion” section by moving part of the text into the “Discussion” section (row 173).

Many thanks for your consideration.

Sincerely,

Dr Andrea Tamburrano

*Section of Hygiene - Institute of Public Health*

*Università Cattolica del Sacro Cuore di Roma*

Dear reviewer #2,

I am thankful for your precious revision made to improve our work. Your suggestions were carefully analysed and, as you can see from the attached reviewed document, completely embraced.

On the basis of your suggestion, we reconsidered the whole paper and the meaning of “inappropriateness” and limited it only to the topic of “test overuse”. Even the title has been changed in this light.

Although it is not feasible to apply appropriateness criteria and/or overutilization rules on all possible laboratory tests (over 1000), according to the other authors mentioned, we considered the most frequently performed tests and representative rules (applicable by automatic systems, considering a CCDSS use). We proceeded to specify this aspect in the “Setting” section (row 88) and to better explain this topic in the “Strengths and limitations” section (row 194).

In this light, as you correctly mentioned, in “Computerized Clinical Decision Support System (CCDSS)” section (row 104) we rephrased the over-utilization criteria specifying the number of biological invariance and incompatibility rules and the preponderance of minimal re-testing intervals criteria. Also, the sentence “The rules, based on the most recent international guidelines on the prescription appropriateness for laboratory tests” was rephrased (row 105).

In Table 1 we provided a better explanation of the rule applied to Alpha Fetoprotein (AFP): “Test cannot be requested with nonspecific tumor markers (CEA, CA125, CA19-9, CA15-3, TPA)”.

As you suggested, we proceeded to explain the context of the Italian National Health System by specifying in the “Analysis” section that all treatments were carried out free of charge for the hospitalized patients; hospitalization costs (including laboratory tests) were reimbursed by the Regional Health System according to Medicare Diagnosis Related Groups (MS-DRGs) (row 132). Italian NHS fully covers hospitalization costs according to MS-DRG. This is a flat cost and it’s linked to the main diagnosis and procedures performed during hospitalization. A reduction in inappropriate laboratory tests does not affect the reimbursement costs and generates net savings for the hospital.

Although your suggestion to consider analytical and secondary costs is acceptable and very interesting, unfortunately, our data does not allow to make a deep economic evaluation. However, in case of over-utilization criteria and minimal retesting intervals, costs due to a prolonged length of stay, unnecessary diagnostic tests and therapies are substantially negligible.

Following your indications, in the “Discussion” section (row 177) we proceeded to better clarify that comparisons with other authors’ results are affected by a reasonable variability in terms of settings and tests considered. However, only comparisons with over-utilization rates of other authors have been included.

According to your suggestion, we have reduced the “Conclusion” and increased the “Discussion” sections.

As you cited, some tests have a lower number of monthly requests (due to particular clinical needs that motivate the request, related to the epidemiology of the population). Nevertheless, over-utilization rates of each exam were calculated on the total of the requests on a period of 20 months. In this case, rates were sufficiently reliable because they were calculated on 3,520 alpha fetoprotein tests and 1,120 chlamydia trachomatis nucleic acid amplifications (NAATs).

Finally, information on hospital’s location (row 83) was added.

Many thanks for your consideration.

Sincerely,

Dr Andrea Tamburrano

*Section of Hygiene - Institute of Public Health*

*Università Cattolica del Sacro Cuore di Roma*
