# Supplementary material for: Evaluation and cost estimation of laboratory test overuse in 43 commonly ordered parameters through a Computerized Clinical Decision Support System (CCDSS) in a large university hospital
Source: PLoS One. 2020 Aug 6;15(8):e0237159. doi: 10.1371/journal.pone.0237159 (PMC7410244; doi:10.1371/journal.pone.0237159)
Supplement: Attachment — Submitted filename: Response to reviewers.docx [file pone.0237159.s003.docx]

**Dear editor,**

I am thankful for your precious suggestions made to improve our work. They were carefully analysed and, as you can see from the attached reviewed document, completely embraced.

Reviewers’ suggestions were now fully addressed, and all the sections of the paper were improved.

Many thanks for your consideration.

Sincerely,

Dr Andrea Tamburrano

*Section of Hygiene - Institute of Public Health*

*Università Cattolica del Sacro Cuore di Roma*

**Dear reviewer #1,**

I am thankful for your precious revision made to improve our work. Your suggestions were carefully analysed and, as you can see from the attached reviewed document, completely embraced.

Following your indications, the “conclusion” section has been shortened and the “discussion section” has been improved.

Many thanks for your consideration.

Sincerely,

Dr Andrea Tamburrano

*Section of Hygiene - Institute of Public Health*

*Università Cattolica del Sacro Cuore di Roma*

**Dear reviewer #2,**

I am thankful for your precious revision made to improve our work. Your suggestions were carefully analysed and, as you can see from the attached reviewed document, completely embraced.

Following your indications, the title was changed, also “discussion” and “conclusions” sections were improved.

We report point-by-point answers to your questions:

*• The mentioned biological invariance rules are basically minimal re-testing interval rules. In the CCDSS-section this should at least be put in brackets behind the term “biological invariance rules” so that the readers are readily able to follow your chain of thoughts.*

As you requested, we put in brackets the term “minimal re-testing intervals” next to “biological invariance rules” (row 100).

*• My suggestion regarding the change of the title was not considered. Once again, I would urge the authors changing it to “selected laboratory tests” or “43 laboratory tests” instead of “laboratory tests”, otherwise readers may assume that all parameters from the labs portfolio were considered in this study, which is not true. Suggestion: “Evaluation and cost estimation of laboratory overuse in 43 parameters by the use of a Computerized Clinical Decision Support System (CCDSS) in a large university hospital.”*

As you reported, we changed the title of the paper, following your suggestion, in “Evaluation and cost estimation of laboratory test overuse in 43 commonly ordered parameters through a Computerized Clinical Decision Support System (CCDSS) in a large university hospital”.

*• The authors misunderstood my suggestion regarding the calculations of costs. Calculating these numbers based on local reimbursement fees may be interesting for the region the study was performed in. However, for all the other readers of PLOS One these numbers are meaningless, since they are not comparable. Therefore, I would suggest a clear and transparent cost calculation based on reagent costs and if possible also personnel and material (blood collection set) costs. As supplemental table a list of single costs for each analyte should be provided. Thereby, readers are able to estimate respective savings in their own setting.*

*Please also consider the publication rules of PLOS One in this context: “The PLOS Data policy requires authors to make all data underlying the findings described in their manuscript fully available without restriction, with rare exception (please refer to the Data Availability Statement in the manuscript PDF file). The data should be provided as part of the manuscript or its supporting information, or deposited to a public repository. If there are restrictions on publicly sharing data—e.g. participant privacy or use of data from a third party—those must be specified.”*

Regarding the calculation of the costs for each laboratory test, we applied the most reliable and independent calculation available within the Italian healthcare system. In fact, the calculation based on the breakdown of the different items and salaries would return an arbitrary evaluation based on the local hospital contracts with companies. Overall the provided estimates are official evaluation from National Italian authorities and therefore provide the best and standardized estimate we may provide to the PLOS ONE readers.

Different authors analysed this issue and drew similar conclusions.

- Irene et al., for example, say:

“*To attach a cost to a single test or a panel of tests is not a straightforward task due to many factors, including the variability between direct and indirect/overhead costs, proprietary information from vendors on how consumables and equipment costs are broken down, service contracts, and the variability of how each clinical laboratory processes their laboratory tests”*

and

*“Although studies have presented “actual costs” of laboratory tests that are hospital- or province-specific, an “actual cost” of performing a laboratory test varies tremendously across hospitals, cities and provinces. Here, we present the reference median costs (RMC) of commonly ordered laboratory tests in a Canadian setting as a first step towards raising physician awareness in order to help enhance selective and appropriate laboratory test ordering practices.*

and

*“Calculating the cost of a laboratory test is complicated as there are a number of fluctuating factors associated with the costs of performing each test sample and the production of the laboratory test result. […] Due to the variability of direct expenses relating to the production of a test result, and variable volumes of tests processed between clinical laboratories, the RMC of each test presented here was determined by compiling price lists of all inclusive indirect costs.”*

- Following the same concept, also Sarkar et al. say:

*“Approximation of financial cost burden was based on test pricing from two commercial laboratories that might not reflect all commercial laboratories. Hence, the cost analysis should be considered only as an estimation.”*

- Finally, Bertrand et al., say:

*The measurement of the direct cost of these inappropriate procedures only included the direct cost of the test. For laboratory tests, each item or group of laboratory items was associated with a fixed value (e.g., 10 CHF for the CRP assay). […] The total cost was therefore obtained by adding the set of values associated with the repeated tests. This cost, therefore, did not take into account indirect costs, such as nursing services, time required to complete exams, equipment costs, etc. […] The costs generated by these repeated procedures were estimated on the basis of the unit rates charged at the HUG at CHF 20,000 over one year.”*

1. Ma, I., Lau, C. K., Ramdas, Z., Jackson, R., & Naugler, C. (2019). Estimated costs of 51 commonly ordered laboratory tests in Canada. Clinical Biochemistry, 65, 58–60. <https://doi.org/10.1016/j.clinbiochem.2018.12.013>
2. Sarkar, M. K., Botz, C. M., & Laposata, M. (2017). An assessment of overutilization and underutilization of laboratory tests by expert physicians in the evaluation of patients for bleeding and thrombotic disorders in clinical context and in real time. Diagnosis, 4(1), 21–26. <https://doi.org/10.1515/dx-2016-0042>
3. Bertrand, J., Fehlmann, C., Grosgurin, O., Sarasin, F., & Kherad, O. (2019). Inappropriateness of Repeated Laboratory and Radiological Tests for Transferred Emergency Department Patients. Journal of Clinical Medicine, 8(9), 1342. https://doi.org/10.3390/jcm8091342

- *I understand the authors stating that deep economic evaluation of secondary costs is not feasible. In addition thereof however, the statement “costs due to a prolonged length of stay, unnecessary diagnostic tests and therapies are substantially negligible.” is just a subjective belief and imho severely false. – this is just a personal statement and does not have to be discussed in the manuscript.*

Following your indications, the statement was reported in the correct formula in the “conclusions” section (row 222-224)

*• My suggestion regarding the discussion section was completely ignored: The discussion is way to short. I would urge the authors to discuss also the patient risk of inappropriate lab testing, which is far more important than costs of testing. Additionally, possible causes for inappropriate testing within your health care setting as well as ways of prevention should be discussed.*

Following your indications, the issues you raised were included and argued in the “discussion” section (row 195-203).

Many thanks for your consideration.

Sincerely,

Dr Andrea Tamburrano

*Section of Hygiene - Institute of Public Health*

*Università Cattolica del Sacro Cuore di Roma*

**Dear reviewer #3,**

I am thankful for your precious revision made to improve our work. Your suggestions were carefully analysed and, as you can see from the attached reviewed document, completely embraced.

Following your indications, the issues you raised were included and argued in the “discussion” section (row 195-203).

As you reported, “ematology” was corrected in “hematology”.

Finally, a description of the descriptive statistics performed is reported in the “analysis” section (row 127-129).

Many thanks for your consideration.

Sincerely,

Dr Andrea Tamburrano

*Section of Hygiene - Institute of Public Health*

*Università Cattolica del Sacro Cuore di Roma*
