# Supplementary material for: Evaluation and cost estimation of laboratory test overuse in 43 commonly ordered parameters through a Computerized Clinical Decision Support System (CCDSS) in a large university hospital
Source: PLoS One. 2020 Aug 6;15(8):e0237159. doi: 10.1371/journal.pone.0237159 (PMC7410244; doi:10.1371/journal.pone.0237159)
Supplement: Attachment — Submitted filename: Response to reviewers.docx [file pone.0237159.s004.docx]

**Dear editor,**

I am thankful for your precious suggestions made to improve our work. They were carefully analysed and, as you can see from the attached reviewed document, completely embraced.

Reviewers’ suggestions were now fully addressed, and all the sections of the paper were improved.

The provider of the *Prometeo Appropriatezza* Software has been provided in parentheses (row 97).

The discussion section has been improved by adding a discussion on patient safety risks related to inappropriate routine tests (row 203-210 and 214-215).

Many thanks for your consideration.

Sincerely,

Dr Andrea Tamburrano

*Section of Hygiene - Institute of Public Health*

*Università Cattolica del Sacro Cuore di Roma*
